# Assessing Neonatal Transport, Health Outcomes, and Psychosocial Inequities Among Black, Very Preterm Infants and Their Families to Advance Health Equity: Protocol for a Mixed Methods Study

**DOI:** 10.2196/84626

**Published:** 2026-04-10

**Authors:** Karen F Warren, Tisha Felder, Nansi Boghossian, Robin M Dawson, Robin B Dail

**Affiliations:** 1 Department of Biobehavioral Health and Nursing Science, College of Nursing, University of South Carolina, 1601 Greene Street, Columbia, SC, 29208, United States, 1 5402572007; 2 Department of Epidemiology and Biostatistics, Arnold School of Public Health, University of South Carolina, Columbia, SC, United States

**Keywords:** neonatal transport, maternal mental health, very preterm infant, health disparities, neonatal intensive care unit

## Abstract

**Background:**

Despite medical advancements, preterm birth rates in the United States remain high and contribute substantially to infant mortality and long-term morbidity, with Black families disproportionately affected. Very preterm (VPT) infants—born between 24 and 30 weeks’ gestation—are frequently delivered at community hospitals that lack advanced neonatal care and therefore require postnatal transport to tertiary neonatal intensive care units (NICUs). While neonatal transport is essential for optimizing infant outcomes, it can create additional challenges for families, including geographic separation from the NICU, disruptions to early parent-infant bonding, and increased parental psychosocial stress. These challenges may be further intensified by social vulnerability and experiences of perceived discrimination in health care settings.

**Objective:**

This study examines associations between neonatal transport, infant health outcomes, and the psychosocial well-being of Black parents with VPT infants. It also examines how structural inequities and social vulnerability shape experiences from delivery through NICU hospitalization.

**Methods:**

This protocol describes a longitudinal mixed methods multiple case study at a single perinatal center in South Carolina. Up to 10 cases of VPT Black infants transported from community hospitals between May 2023 and January 2024 are included. Each case includes the infant and mother, with partners invited. Quantitative data include infant outcomes from electronic medical records, neighborhood disadvantage using the Area Deprivation Index, and parent surveys of stress, anxiety, depression, bonding, perceived discrimination, and access to care. Qualitative data come from semistructured interviews guided by an adaptation of the National Institute on Minority Health and Health Disparities Research Framework and analyzed using reflexive thematic analysis. Data are integrated within each case to create structured summaries, followed by cross-case comparisons to identify patterns and variation.

**Results:**

The study was funded by the National Institute of Nursing Research in July 2023. Participant enrollment occurred between May 2023 and January 2024, with 6 cases enrolled. Data collection and electronic medical record abstraction were completed in May 2024, with analysis completed by July 2024. This manuscript reports the protocol and analytic plan. Detailed results will be reported in 2 companion manuscripts currently under review; publication of one is anticipated in fall 2026.

**Conclusions:**

This protocol outlines a novel mixed methods case study of neonatal transport and infant and parent outcomes among Black families. The study is exploratory and intended to generate hypotheses and inform future larger scale research and intervention development to support parental mental health, reduce disparities, and improve care experiences following preterm birth and neonatal transport.

## Introduction

### Background

Despite medical advancements, global preterm birth rates have remained largely unchanged over the past decade [1]. As survival rates of preterm infants have improved [2], neonatal intensive care unit (NICU) admissions and length of stay have increased [3]. Preterm birth—defined as delivery before 37 weeks of completed gestation—remains a leading cause of neonatal mortality and lifelong morbidity worldwide [4]. Prematurity and low birth weight are the second-leading causes of infant mortality in the United States and major contributors to both short- and long-term morbidities, including respiratory, neurodevelopmental, and behavioral complications [4]. Annually, more than 70,000 infants are transported from community hospitals to NICUs to manage complications related to preterm birth [5].

While neonatal transport is essential for optimizing infant outcomes, it often creates additional challenges for families, especially those living far from the NICU. Parents of very preterm (VPT) infants—particularly those from low socioeconomic backgrounds—experience disproportionately high levels of psychological distress, including elevated levels of stress, anxiety, depression, and posttraumatic stress symptoms [6-9]. Recent evidence suggests that 30% to 50% of parents of preterm infants experience clinically significant mental health symptoms, which may persist for months to years after discharge [10,11].

Families with fewer socioeconomic resources or those living far from tertiary NICUs face additional barriers, including transportation costs, missed employment, childcare challenges for siblings, and limited access to their infant [12,13]. These stressors are associated with higher rates of depression and psychological distress among parents, particularly those from historically marginalized communities [7,14].

Elevated parental stress and depression have been linked to reduced participation in NICU care, altered parent-infant bonding, and adverse neurodevelopmental and behavioral outcomes that extend into early childhood [15]. Racial inequities in parental stress and mental health outcomes persist, with Black parents of preterm infants reporting higher levels of stress, depression, and perceived discrimination in health care settings, even after accounting for illness severity in their infant and socioeconomic status [16]. Structural inequities in access to high-quality perinatal care and mental health care may further compound these challenges. Despite this growing body of literature, no studies have examined how neonatal transport specifically contributes to parental psychosocial outcomes among Black families.

To contextualize these disparities, this study is informed by the National Institute on Minority Health and Health Disparities (NIMHD) Research Framework, which conceptualizes health outcomes as the result of interacting influences across multiple domains. These domains (biological, behavioral, sociocultural, and health care system factors) operate at the individual, interpersonal, community, and societal levels. Applying this framework allows for the examination of how multilevel determinants contribute to inequalities experienced during birth, transport, and NICU hospitalization.

### Study Aims

#### Aim 1: Examining the Impact of Neonatal Transport on Infant Health Outcomes

Pathways resulting in poor infant outcomes are often complex and multifactorial. Community hospitals and staff vary greatly in their resources to stabilize and resuscitate VPT infants. Birth in community hospitals has been associated with increased morbidity such as hypothermia, severe intraventricular hemorrhage, chronic lung disease, and other poor outcomes resulting from physical stress and lifesaving procedures [17-19]. We aim to examine cases for conditions that may have an association with the stabilization trajectory at the birth hospital or during transport, which may negatively affect an infant’s functioning or quality of life and pose challenges and costs to families. Studies have shown that VPT infants born in a community hospital and requiring transport have significantly higher odds of morbidities, cognitive impairment, and mortality risk [20-27]. Morbidities of interest known to be higher in the VPT-transported infant population were selected for data collection during NICU hospitalization. Any additional described morbidities were also noted to contextualize each case. Long-term health outcomes are less than optimal for this population, as multiple studies reveal that infants requiring transport have a higher chance of developmental impairment, cerebral palsy, and overall higher odds of death than those born in facilities with optimal capabilities [20-22,28].

#### Aim 2: Examining Parent Psychosocial Health, Social Vulnerability, and Socioeconomic or Racial Inequities

In the United States, Black women are more likely to deliver preterm infants than White women [29]. Mothers of VPT infants are more likely to live in areas with higher social vulnerability [30]. Further exacerbating these resource issues, VPT infants have considerably higher costs and use more health care resources across all health care service categories than their full-term peers during their first year of life [31]. Psychological morbidities in parents are common following childbirth, with depressive symptoms estimated to be 4.5% to 20% in mothers and 3% to 10% in fathers [32-34]. Research has shown that one partner’s depressive symptoms can negatively affect the other partner’s ability to bond with their child and that stress experienced by one parent often contributes to increased stress in the other [33,35]. Distance from the parents’ home to the hospital NICU is another significant stress indicator [12], also affecting parent-infant bonding and further compounding stress and anxiety or leading to depression. Because structural racism in the health care system may also play an important role in racial disparities [36], we aim to investigate parents’ perceptions of bias and racism in relation to neonatal transport and NICU hospitalization.

The purpose of this paper is to report a study protocol designed to accomplish the stated aims by examining the association between neonatal transport, the health outcomes of Black VPT infants, and the psychosocial well-being of their parents, with particular attention to social vulnerability, and socioeconomic and racial inequities experienced during birth, transport, and NICU hospitalization. A deeper understanding of these relationships is crucial for improving access to individualized, high-quality care and for advancing health equity.

## Methods

### Study Setting

The study was conducted in South Carolina, where the preterm birth rate for Black women is 55% higher than that for other racial and ethnic groups, and the infant mortality rate exceeds the national average [1,37]. Recruitment took place at a tertiary level 3 NICU at a local health system in a medium-sized Southeastern US city. This health system includes a children’s hospital and serves as the region’s perinatal center, which covers 11 community hospitals and 16 counties, all of which are ranked moderate to high on the Centers for Disease Control and Prevention Social Vulnerability Index [38]. The most distant site in this region is 1.2 hours away by ambulance. Each case starts at the community hospital where the preterm infant is born. The perinatal center has a specialized neonatal and pediatric critical care transport team stationed onsite, which is responsible for all infant and pediatric patients returning to this center, resulting in more than 300 infant transports annually. Transport may occur by specialized critical care ambulance or by helicopter, depending on the distance and the illness severity of the infant, making the transport vehicle the next setting of the case. The final case setting is the only tertiary level III NICU in the region, with 70 beds and more than 800 infants delivered annually. The NICU manager and the neonatal medical director both provided letters of support for this study.

### Study Design

This descriptive, exploratory study used a multiple-case study design and a mixed methods approach to describe the trajectory of Black preterm infants and their families from birth at a community hospital through transport to a referral center and subsequent NICU hospitalization. A case study design allows for a longitudinal understanding of complex and dynamic processes from multiple perspectives and contexts over time [37] and enables the description, explanation, or exploration of everyday phenomena in their natural settings [39]. Case study research is appropriate for descriptive and exploratory studies that investigate research questions that have not been studied in depth previously [40]. Multiple data collection methods provide a detailed investigation and analysis of the context and processes involved in the phenomenon under study. An adapted version of the NIMHD Research Framework [41] informed the research design and methods [42].

### Ethical Considerations

This study was approved by the Prisma Health Midlands Institutional Review Board (1999826-1). All collected materials, including interviews, medical record information, and surveys, were deidentified and entered into a password-protected database stored on a secure server to protect participant privacy. Informed consent was obtained from all participants, who were informed that they could withdraw from the study at any time without consequence. Phone numbers and email addresses were collected at consent. Participants received financial compensation of US $25 for each set of surveys or interviews completed to acknowledge their time and reduce attrition.

### Sample

The study was conducted between May 2023 and June 2024. Each case consisted of the anchor infant: a Black VPT infant born between 24 and 30 weeks’ gestational age (GA), transferred to the NICU, and the infant’s mother, with an invitation to the other parent to participate. Our aim was to enroll 10 infant cases using purposive convenience sampling with deliberate stratified sampling to ensure a range of GAs across infant cases. The sample size was set at 10 to achieve the purpose of the study and to allow for an in-depth examination. In case study methodology, sample size is not determined by statistical power but by the depth, richness, and purpose of the cases, with an emphasis on understanding complex phenomena within a real-world context [43]. Multiple-case study designs with 6 to 10 cases are recommended as sufficient to enable cross-case comparison while maintaining feasibility [44,45]. The intensive data collection also necessitates a limited number of cases to maintain rigor. Importantly, the purpose of this study is not to generalize findings but to generate grounded insights that can inform hypothesis development and intervention design to guide future larger-scale studies.

### Study Procedures

This project was supported by the National Institutes of Health–National Institute of Nursing Research award (1F31NR020731-01A), awarded in July 2023. Institutional review board approval was obtained in advance from the hospital where the research was conducted. The NICU charge nurse notified the research team of any new admissions that met the inclusion criteria and provided contact information. Mothers provided informed consent at the infant’s bedside, if available, or via phone, both for themselves and on behalf of their infants. Inclusion criteria consisted of Black mothers and their infants born between 24 and 30 weeks’ gestation. The other parent was also invited to participate. “Parent” was defined as the other person who would be actively participating in the infant’s care upon discharge. Eligible infants had to be delivered at an outside hospital and transported to the NICU, where recruitment took place. Infants with significant congenital anomalies and mothers who could not speak English were excluded. English-speaking participants were approached because the research team conducting the qualitative interviews was English speaking.

To achieve the objectives of aim 1, we used publicly available information to gather characteristics of the birth hospital. The first author (KFW) performed an electronic medical record (EMR) data review to provide a complete picture of the infant’s transport trajectory. The transport team provided records from the birth hospital and an admission summary that informed the case on stabilization procedures that were given immediately after birth. A thorough review of EMR data from birth through 36 weeks’ corrected GA was conducted for any morbid health outcomes that may have a critical impact on the health and well-being of each infant and their family.

**Figure 1.**
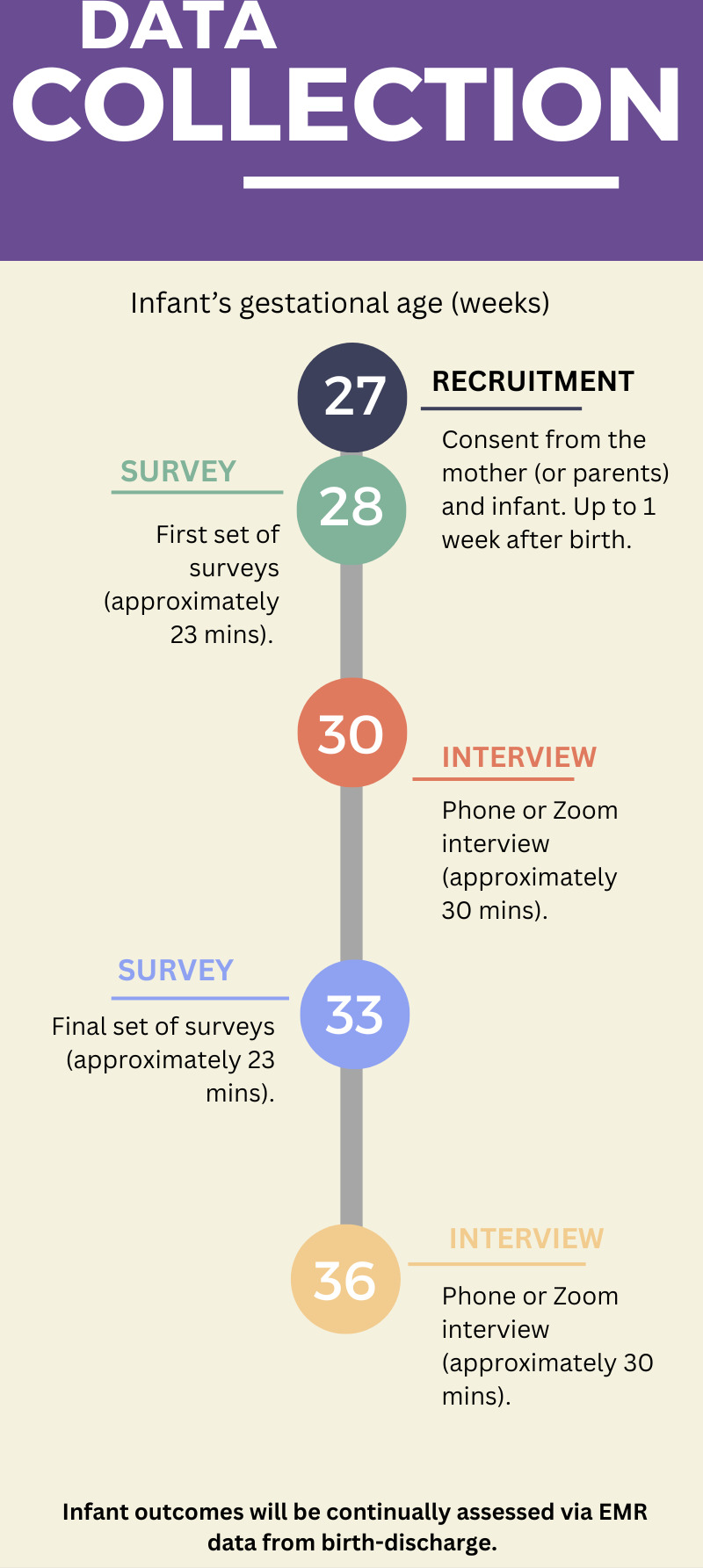
Data collection timeline example. EMR: electronic medical record.

To accomplish the goals of aim 2, parents completed a series of surveys (Table 1). An email was sent with a direct link to a set of surveys on Research Electronic Data Capture (REDCap), which is a Health Insurance Portability and Accountability Act (HIPAA)–compliant web-based application created to capture data for clinical research and to create databases and projects [46]. These surveys were previously published and validated instruments, apart from the infant access questionnaire, which was developed by the study team. Sets of surveys were administered 2 to 3 times (depending on the infant’s length of stay) to measure how responses changed over the course of NICU admission and to understand experiences across multiple phases (refer to Figure 1 for example). The average time to complete an entire set of surveys was 23 minutes. Infant data collected from the EMR by the first author included infant GA, gender, and home address. This address was used to obtain the Area Deprivation Index (ADI) on the Neighborhood Atlas website (a scientifically validated measure that shows socioeconomic disadvantage by neighborhood) [47,48], which was added to REDCap. The ADI is composed of 17 measures related to education, employment, housing quality, and poverty, originally drawn from long-form census data and updated to incorporate more recent American Community Survey data [47,48]. Scores within a state are ranked from lowest to highest and then divided into deciles (1 to 10), with 10 representing the most disadvantaged neighborhood groups. Research shows that socioeconomic status can be a fundamental cause of health disparities [49].

**Table 1.** Study outcomes and corresponding measures.

Expected outcomes	Measure	Description
Parental bonding	Parent-to-Infant Attachment Questionnaire [50]	It is a 25-item questionnaire using a 6-point Likert scale to assess the occurrence of disturbances in postpartum bond formation between mother and child.
Barriers to infant access	Team created a survey	It is an 8-item survey assessing descriptive factors (distance and cost of travel, transportation and accommodation, and visitation barriers or hardships).
Parental perceptions of bias and discrimination—health care experience	Discrimination in Medical Settings [51]	It is a 7-item instrument using a 5-point Likert scale to assess previous experiences of mistreatment in health care attributed to race, ancestry, or national origin.
Parental perceptions of bias and discrimination—comparative experience	Single question from Behavioral Risk Factor Surveillance System [52]	It assesses whether, in the past 12 months, health care experiences were worse than, the same as, or better than those of individuals of other races.
Parental perception of free choice to make informed health care choices	Adapted Medical Outcomes Study [53]	It is a measure that asks: (1) If there were a choice between treatments, how often would your child’s doctor ask you to help make the decision? (2) How often does your child’s doctor give you some control over your treatment? (3) How often does your child’s doctor ask you to take some of the responsibility for your treatment?
Parental psychosocial morbidities—stress	Parental Stress Scale: Neonatal Intensive Care Unit [54]	It is a 26-item questionnaire assessed with a 5-point Likert scale to quantify parental stress in the particular situation of preterm birth.
Parental psychosocial morbidities—anxiety	Beck Anxiety Inventory [55]	It is a 21-item self-report questionnaire using 4-point scale to assess the intensity of physical and cognitive anxiety symptoms during the past week.
Parental psychosocial morbidities—depression	Edinburgh Postnatal Depression Scale [56]	It is a 10-item questionnaire using a 4-point Likert scale to assess depressive symptoms and coping related to pregnancy and childbirth
Socioeconomic status and disadvantage	Survey and Area Deprivation Index [57]	It is a multiple-choice 8-item survey assessing descriptive characteristics (age, race, gender, marital status, education, paid leave, occupation, annual household income, and insurance status) and Area Deprivation Index rank for social disadvantage.

To gain deeper insight into the psychosocial health of mothers—including their experiences during pregnancy, of having a preterm infant, and of navigating neonatal transport—the study team developed semistructured interview guides informed by key constructs from an integrated theoretical framework. This framework is an adapted version of the National Institutes of Health’s NIMHD Research Framework, which is described in detail in a separate publication [42]. Probing questions were used to elicit deeper reflections on emotional support, staff interactions, perceived needs, and related experiences. Sample questions from the interview guide are presented in Table 2. To maximize participation, these interviews were conducted by phone or via Zoom teleconferencing.

**Table 2.** Sample interview questions.

Interview question	Additional probing example
Tell me about your pregnancy experience over the past __ months and what really stands out to you about this time in your life.	Who is the person or people you feel gave you emotional support during your pregnancy?
Tell me about your delivery experience with your baby at __ hospital.	Are there any parts of your labor and delivery experience at the hospital that stand out to you, either good or bad?
The transport team brought your baby to this hospital. Did someone talk with you before your baby was taken away in the ambulance?	Tell me any ways the process of taking your baby to another hospital might have been better for you.

### Data Analysis

#### Overview

The case study approach allows for an in-depth, multifaceted examination of an underresearched phenomenon in a real-life setting. Case study methodology integrates well with mixed methods, which seeks a more complete understanding through the integration of qualitative and quantitative research [37]. We chose this approach to address both aims across the trajectory of the case. This multiple-case, longitudinal, exploratory study, with within-case and across-case analyses, used a personalized, concurrent mixed methods approach with repeated data collection points, as parental experiences are shaped by the unique trajectory of their infant’s NICU experience and their own experiences within the NICU.

#### Quantitative Analysis

Three types of quantitative data were collected: (1) surveys administered to the parents at sequential time points for variables of interest, (2) the ADI to measure neighborhood disadvantage, and (3) infant morbidity outcomes from the EMR. All data, including survey data, were uploaded into REDCap as research capture tools. Because this study is exploratory and not powered for inference due to the small sample size, we used descriptive statistics (including means, SDs, and ranges) to analyze and summarize all data for each case. These findings were used to characterize each case, summarize patterns, and support cross-case comparisons. The primary purpose of this study was to generate hypotheses rather than test hypotheses. By describing infant clinical trajectories and parental psychosocial experiences, this study aims to identify relationships that may be examined in future studies with larger samples.

#### Qualitative Analysis

Research questions used for semistructured interviews were guided by an adapted model of the NIMHD Research Framework [58]. Parent interviews were recorded, typically lasting 20 to 40 minutes. Transcription was performed by the first author and was reviewed and compared to the recordings to ensure that they represent participants’ utterances as accurately as possible. Reflexive thematic analysis, as outlined by Braun and Clarke [59], was used to explore the experiences of Black mothers navigating the emotional journey of giving birth to a VPT infant who required both neonatal transport and NICU hospitalization. This robust 6-phase framework includes (1) familiarization with the data, (2) generating initial codes, (3) searching for themes, (4) reviewing themes, (5) defining and naming themes, and (6) writing the report [60]. The inductive approach was used, with no attempt to fit the data into a researcher’s preconceptions [61]. Themes and subthemes were identified by the first author, RMD, and RBD.

After quantitative and qualitative data analyses, data were integrated to comprehensively summarize each enrolled case and reviewed for deviations from the interpretive summary by the first author and RBD. Each structured case summary included the infant’s clinical course (with data spanning from birth at a community hospital until NICU discharge), longitudinal parental psychosocial measures, and themes derived from parent interviews. Quantitative data were summarized descriptively. Data were then applied to a visual timeline of the trajectory, comparing the infant’s clinical status to the parent’s psychosocial health to enhance interpretation. Cross-case analyses explored commonalities and divergences in participants’ aggregate experiences. After case summarization, between-case comparisons were discussed by the entire research team. These results will be used to generate hypotheses for future studies.

### Reflexivity

The first author was a novice qualitative researcher. She was a doctoral student studying in the field of nursing science, with an interest in preterm infant outcomes and maternal experiences. Her clinical background, spanning almost 2 decades, was in neonatal critical care, both in the NICU and as a NICU or pediatric ICU flight nurse. The familiar context provided benefits such as access and a working knowledge of both NICU and neonatal transport policies and procedures. Awareness of positionality was vital, as she identifies as a White researcher interviewing Black women. Particular attention was given to how racial and social position may have shaped the dynamics and data interpretation. Efforts were made to build trust through transparency about the study’s purpose, active listening, and validation of participants’ experiences, while recognizing that trust is not assumed but earned. Notes were taken throughout the analysis to document assumptions and emotional responses and to examine how the first author’s positionality may have influenced theme interpretation and development. Discussions with coauthors were used to further interpret the data and enhance reflexive rigor.

To support methodological rigor, training in qualitative data collection and analysis was provided by the methodological expert (RMD) to the first author, as well as to members of the research team prior to study initiation and reinforced at intervals throughout the study. The intention was for author KFW to develop the knowledge and skills to be an independent researcher. Author RMD is a faculty member at the study university with expertise in qualitative methods and research focused on communication processes in vulnerable and underserved populations and their contributions to health disparities. Authors KFW and RMD each conducted independent reflexive thematic analysis of the interview data. These interpretations and themes were used to enhance depth rather than achieve consensus.

## Results

This study examined the relationships between neonatal transport and both infant health outcomes and parental psychosocial well-being among Black families with VPT infants. Participant enrollment occurred between May 2023 and January 2024, during which time 6 cases were enrolled. During the enrollment period, neonatal transport admissions were reduced following the implementation of a high-risk maternal transport program, and all eligible cases that met the inclusion criteria were recruited. All enrolled cases were included in the analysis to inform the interpretation of findings.

Study activities, including EMR review and abstraction across NICU hospitalization, survey administration, and interviews, were completed in May 2024, with analysis completed by July 2024. Data collection was comprehensive and encompassed detailed clinical information, parental psychosocial measures, and qualitative data necessary to address the study aims. Detailed findings from this study will be reported separately in 2 companion manuscripts: one presenting overall study results with an illustrative case exemplar and another focusing on the qualitative results of parental psychosocial outcomes. Portions of this work have also been presented at regional, national, and international conferences.

## Discussion

### Important Implications for Practice

Health systems and social service networks have a significant opportunity to mitigate the psychosocial challenges experienced by NICU mothers in the postpartum period by providing avenues of support to help mothers cope with the transition process. Guidelines have been proposed to support the integration of psychologists into NICU settings, and a growing network of NICU psychologists has emerged [62]. There is growing evidence that interventions can improve parental mental well-being in the NICU; however, further research is needed to evaluate emerging technology-based and telehealth approaches [63,64].

Previous literature has highlighted the role of structural factors (such as limited access to postpartum mental health services, communication challenges within health care systems, and perceived discrimination) in shaping maternal experiences and contributing to inequitable outcomes [10,65]. Provider education addressing bias and increasing awareness about the barriers faced by Black mothers can reduce issues perpetuated within systems. Legislative and policy solutions to improve maternal access and quality of care and to sustain funding for community-based maternal health programs could improve future outcomes for Black women and infants.

### Limitations

Eligibility for this study was limited to a small population of Black mothers who delivered a preterm infant between 24 and 30 weeks’ GA at a community hospital and required transport to a NICU. Recruitment of mothers for this study was within a single perinatal center in the southern United States; therefore, the experiences of mothers in other regions may be different.

### Conclusions

This multiple-case study design and mixed methods approach provides exploratory insight into the real-life experiences of Black mothers following preterm birth and neonatal transport. To our knowledge, this is the first study to examine the experiences of Black women who delivered a preterm infant in a community hospital that required neonatal transport, using a mixed methods case study lens. Through intensive, case-level data collection, the protocol outlines an approach for capturing maternal perspectives, which can highlight the challenges and systemic barriers present within our health care system across all phases, from pregnancy to several months postpartum. This protocol outlines a small foundational study designed to generate hypotheses and inform future larger-scale research. The findings generated from this study will be used to guide the development of subsequent research aimed at supporting mothers during this challenging and stressful period, tailoring interventions to reduce disparities, and improving maternal mental health outcomes.

## Supplementary material

10.2196/84626 Peer Review Report 1 National Institutes of Health–National Institute of Nursing Research F31 Summary Sheets

## Abbreviations

ADI: Area Deprivation Index
EMR: electronic medical record
GA: gestational age
HIPAA: Health Insurance Portability and Accountability Act
NICU: neonatal intensive care unit
NIMHD: National Institute on Minority Health and Health Disparities
REDCap: Research Electronic Data Capture
VPT: very preterm
