# Supplementary material for: Assessing Neonatal Transport, Health Outcomes, and Psychosocial Inequities Among Black, Very Preterm Infants and Their Families to Advance Health Equity: Protocol for a Mixed Methods Study
Source: JMIR Res Protoc. 2026 Apr 10;15:e84626. doi: 10.2196/84626 (PMC13068191; doi:10.2196/84626)
Supplement: Peer Review Report 1 [file resprot-v15-e84626-s001.pdf]

SUMMARY STATEMENT
(Privileged Communication)

PROGRAM CONTACT:
Dr. Dionne Godette
dionne.godette@nih.gov

Release Date: **03/24/2023**
Revised Date:

Application Number: **1 F31 NR020731-01A1**
Formerly: **1F31NR020731-01**

WARREN,KAREN
University of South Carolina
1601 Greene Street
Columbia, SC 292084001

Review Group: **NRRC (86)**
National Institute of Nursing Research Initial Review Group

Meeting Date: **02/23/2023**
Council: **MAY 2023** ***PCC:*** **CPHDG**
Requested Start: **07/01/2023**
Dual IC(s): **HD**

Project Title: **Exploring the impact of neonatal transport on the health of Black preterm infants and psychosocial challenges faced by parents in relation to social determinants of socioeconomic and racial inequities**
Requested**: 1 Year**

Sponsor: **Dail, Robin B**
Department: **College of Nursing**
Organization: **UNIVERSITY OF SOUTH CAROLINA AT COLUMBIA**
City, State: **COLUMBIA SOUTH CAROLINA**

SRG Action: **Impact Score:30**
Next Steps: **Visit https://grants.nih.gov/grants/next_steps.htm**
Human Subjects: **30-Human subjects involved - Certified, no SRG concerns**
Animal Subjects: **10-No live vertebrate animals involved for competing appl.**
Gender: **1A-Both genders, scientifically acceptable**
Minority: **1A-Minorities and non-minorities, scientifically acceptable**
Age: **1A-Children, Adults, Older Adults, scientifically acceptable**

1F31NR020731-01A1 Warren, Karen

RESUME AND SUMMARY OF DISCUSSION: This report summarizes the scientific review meeting discussion of a resubmitted F31 Predoctoral Fellowship application submitted by Ms. Karen Warren from the University of South Carolina at Columbia requesting for one year of support. Preterm infant death rates are known to be disproportionately higher in Black mothers/infants in comparison to their White counterparts. The first two aims of this application are to assess the impacts of neonatal transport, neonatal intensive care unit (NICU) hospitalizations of Black preterm infants on the health outcomes of their parents and evaluate the longitudinal clinical trajectory, morbidity rate of those infants. The third aim is to identify the barriers, and psychosocial challenges faced by the parents related to these difficulties. Strengths of this application include an applicant who is a neonatal nurse and has extensive clinical experience in the proposed area of research. Letters of recommendation and support are strong. She has a close working relationship with her sponsor. She has assembled a multidisciplinary mentoring team and relevant expertise will be provided. The research training plan is significant pertaining to health disparities in higher preterm morbidity and mortality in Black newborns, and social determinants of health (SDOH) of newborn transportation to hospitals. A case-based study design and mixed methods approach will be utilized, and important data related to Black infants and their parents will be collected. Strong institutional commitment is provided. The resubmission is also viewed as highly responsive to the previous critiques by focusing on Black families only in the proposed study. Weaknesses include the applicant's past academic performance is somewhat mixed; however, her current grades are outstanding. The surveys and interview guides to be used in this study are non-specific; and questions related to Black preterm morbidities are not included. Impacts of maternal breast milk use, a potentially important factor on preterm mortalities and several other lethal conditions are not addressed. It is unclear if both parents will be interviewed at the same time and too many interviews are planned. The training plan mainly focuses on mentorships, and concerns are that little formal coursework is proposed. The training timeline is short and ambitious; the feasibility of finishing of both study and training in such a short period is questioned. Although the stellar applicant, highly significant research topic related to racial disparities, an outstanding environment, and high responsiveness in the resubmission represent as the strengths, the weaknesses observed in the research training plan somewhat dampen the overall enthusiasm. The potential of this fellowship training application is moderately high, and this application received an excellent overall impact score for its scientific and technical merit.

DESCRIPTION (provided by applicant): The United States (US) is one of the ten countries in the world with the greatest number of preterm births. Prematurity and low birth weight are the second leading causes of infant deaths in the US and the leading cause of short and long-term morbidities. In 2020, the rate of preterm single births was almost 65% higher for Black mothers compared to White. For every 1000 preterm or low birthweight infants born, 25 more Black infants die than their White peers. Social Determinants of Health (SDOH) can affect infant outcomes. Individuals of different racial backgrounds and depressed socioeconomic status (SES) are at most risk for lack of access to health care and often receive inadequate care and deliver their infants at lower- quality hospitals than their White counterparts, contributing to higher rates of preterm birth among these groups. Annually, over 70,000 infants require transport to Neonatal Intensive Care Units (NICUs) to prevent and manage complications due to preterm birth. Once an infant is transported, this presents an added challenge to parents, whose home may be of distance from the NICU. Parental stress is high, and families of low socioeconomic backgrounds may face additional challenges such as financial burdens, higher depression rates and limited access to their infant. Understanding the barriers and psychosocial challenges faced by parents due to transport and NICU hospitalization of their infant in relationship to social determinants of socioeconomic and racial inequities is integral to reducing health disparities and improving the quality of life and well-being of the next generation. The proposed research supports the

National Institute of Nursing Research's strategic plan to support studies aimed at prevention and health promotion targeting elimination of health disparities, fostering health equity, and considering social determinants of health (SDOH) to mitigate risks to families of preterm infants. Research Aims: Aim 1: Describe each infant's transport trajectory, including baseline characteristics of the community hospital of birth (size, level of care, birthing resources), transport characteristics (mode, distance, personnel), and infant health from birth to arrival of regional referral neonatal intensive care unit. Aim 2: Describe each infant's longitudinal clinical course trajectory including major morbidity from admission to the NICU until 36 weeks corrected gestational age or discharge (whichever is first). Aim 3: Examine and describe the barriers and psychosocial challenges faced by parents due to transport and NICU hospitalization of their infant in relationship to social determinants of socioeconomic and racial inequities. Methods: This descriptive, exploratory study will use a multiple-case study design and mixed methods approach to describe the trajectory of Black out born infants and their families from birth, through transport to a referral center and through a minimum of 6 weeks of NICU hospitalization, for a detailed analysis of each case. Significance: There is an ongoing need for innovative research methods, and new approaches for identifying aspects of structural discrimination and its impact on health disparities and health outcomes.

PUBLIC HEALTH RELEVANCE: This study uses an exploratory, multiple-case study design to examine the health and well-being of 10 Black premature infants and their families after each infant's premature birth at a community hospital in South Carolina and subsequent transport to a neonatal intensive care unit. Our aim is to describe the impact of neonatal transport and NICU hospitalization on the infant's overall health trajectory. We further hope to describe the psychosocial challenges that transport has added to the infant's family, as well as the relationship to social determinants of socioeconomic and racial inequities.

CRITIQUE 1

Fellowship Applicant:	1
Sponsors, Collaborators, and Consultants:	1
Research Training Plan:	3
Training Potential:	3
Institutional Environment & Commitment to Training:	1

Overall Impact/Merit: This revised F31 application is from a strong applicant with extensive clinical experience which provides her with an in-depth understanding of neonatal transport and potential risks to infant and family. In addition, letters of reference and sponsor statements support her excellence as an applicant and are based on prolonged and direct observation of her research potential. The applicant's highly qualified multidisciplinary team are committed to her success, have a strong working relationship with the applicant, and have provided plans for regular mentoring. In addition, the team have the relevant expertise to ensure the project's success. The application focuses on an important understudied problem regarding racial disparities in preterm birth rate and morbidity, the influence of SDoH on transported Black infant health, and the impact of both on family experiences which may provide preliminary data for future intervention related studies. Her plans to evaluate study procedures prior to study initiation will provide important information to increase likelihood of success. In addition, she has completed independent studies regarding neonatal transport and related outcomes which she has presented at an international conference. Significant weakness in the training plan is apparent including lack of information regarding how the surveys and interview guide are created, no consideration of differences in survey and interview participation, and limited inclusion of specific neonatal morbidities known to be higher in Black infants. Furthermore, plans to collect information regarding provision of maternal breast milk are not described and incentives to decrease subject

burden have not been secured. There is strong institutional support for the applicant including letters of support ensuring her ability to recruit from the study NICU. While the team will likely provide excellent training and education as a step towards preparing this applicant towards her goal of becoming an independent researcher, weaknesses in the training plan somewhat limit the training potential of the applicant.

1. Fellowship Applicant

Strengths

- Ms. Warren is a strong applicant with extensive neonatal nursing and transport experience providing her with in-depth understanding of the procedures of neonatal transport including potential risk to infant and family.
- Letters of reference and sponsor statements support her excellence as an applicant.
- The applicant has worked in Dr. Dail's laboratory for two years providing evidence upon which she based her letter of support.

Weaknesses

- Minor: Marginal undergraduate grades which have vastly improved in her PhD studies.

2. Sponsors, Collaborators, and Consultants

Strengths

- The applicant has assembled a multidisciplinary team of highly qualified researchers that based upon their letters of support and statements, are committed to her success.
- Evidence of strong working relationship with Dr. Dail.
- The team is well funded, have a strong publication history and have the relevant expertise to ensure the project success.

Weaknesses

- None noted.

3. Research Training Plan

Strengths

- This application focuses on an important understudied problem regarding racial disparities in preterm birth rate and morbidity, the influence of SDoH on transported Black infant health, and the impact of both on family experiences.
- Successful completion of the project will provide important hypothesis generating information to use for future interventional studies.
- Plans to evaluate feasibility on a single case prior to this F31 funding which will provide valuable information regarding potential refinement of surveys, interviews and procedures.
- Has completed two independent studies with Dr. Dahl to increase her understanding of neonatal transport and related outcomes and has presented this work at an international conference.

Weaknesses

- No information regarding how the surveys and interview guide were created is provided.

- While mentoring activities regarding SDoH, mental health, and racial disparities are included in the training plan, additional formalized coursework is needed.
- Subject burden continues to be a concern. To meet the previous reviewer's concern, incentives are added for interview/survey completion.
- Black mothers are less likely to provide breast milk to their preterm infants and mother's own breast milk is known to reduce prematurity related morbidities, but information related to breast milk consumption is not included.
- Lack of clarity regarding participation in interview/surveys. For example, multiple iteration of participation could occur (all mothers, all fathers, all both, various combinations) as well plans for dealing with missing surveys/interviews.
- Definition of "any morbid health outcome" is vague. While the incidence of intraventricular hemorrhages (IVH) and chronic lung disease (CLD) have been specifically mentioned, other common morbidities including necrotizing enterocolitis and retinopathy of prematurity known to be higher in transported and Black infants are not specifically included.

4. Training Potential

Strengths

- Strength of the applicant and her mentorship team increases probability that she will receive excellent training and education for preparation as an independent and productive researcher.
- If successfully completed, the project will provide a basis for the applicant to pursue future interventional studies.

Weaknesses

- Weaknesses in the training plan somewhat limit the training potential of the applicant.

5. Institutional Environment & Commitment to Training

Strengths

- Strong institutional support from USC's College of Nursing.
- The Perinatal, Pediatrics, & Family Research Center directed by Dr. Robin Dail will provide additional training and education related to at risk families and infants.
- Letters of support from medicine and nursing at the recruitment NICU assure the applicant's ability to recruit.

Weaknesses

- None noted

Protections for Human Subjects: Acceptable Risks and Adequate Protections

- An acceptable plan has been described

Data and Safety Monitoring Plan (Applicable for Clinical Trials Only): Not Applicable (No Clinical Trials)

Inclusion Plans

- Sex/Gender: Distribution justified scientifically

- Race/Ethnicity: Distribution justified scientifically
- For NIH-Defined Phase III trials, Plans for valid design and analysis: Not applicable
- Inclusion/Exclusion Based on Age: Distribution justified scientifically

Vertebrate Animals: Not Applicable (No Vertebrate Animals)

Biohazards: Not Applicable (No Biohazards)

Resubmission

- Most of the previous reviewer comments have been addressed.

Training in the Responsible Conduct of Research: Acceptable

Comments on Format (Required):

- Training will be through courses and one on one mentoring and discussion

Comments on Subject Matter (Required):

- All required content has been included

Comments on Faculty Participation (Required):

- Faculty will participate in one-on-one mentoring and discussion

Comments on Duration (Required):

- Duration is appropriate

Comments on Frequency (Required):

- Frequency is appropriate

Resource Sharing Plans: Not Applicable (No Relevant Resources)

Authentication of Key Biological and/or Chemical Resources: Not Applicable (No Relevant Resources)

Budget and Period of Support: Recommend as Requested

CRITIQUE 2

Fellowship Applicant:	1
Sponsors, Collaborators, and Consultants:	1
Research Training Plan:	3
Training Potential:	2
Institutional Environment & Commitment to Training:	1

Overall Impact/Merit: This is a resubmission that has been very responsive to the previous critiques with major changes in the research design as recommended. The applicant has completed coursework in the PhD program and has a strong experiential base in neonatal transport that informs her study. Her

advisors and mentors are experienced and well aligned to support the proposed work. The case based and mixed methods approach will yield rich data about 10 out born Black neonates and their parents who have experienced transport to a neonatal center for care. It is likely that these data will provide support for further independent work by this student after she completes the PhD. The study involves collecting data with a large number of instruments and a structured interview. There is individualized training and a Spring short course in mixed methods, but completion of all data collection and analysis and defense in one year seems optimistic. A weakness is the subject burden and the potential difficulty in accessing parents' completions of the multiple instruments planned in the study and for the planned structured interviews, as the applicant notes that parents often have difficulties and lack resources to travel to the NICU. The timeline assumes completion of the study between 12/2023 and 5/2024, so availability of funding for this work would be just available for data analysis and defense.

1. Fellowship Applicant

Strengths

- This student is acknowledged by her mentors to be an excellent student who is preparing for a career as an independent nurse scientist.
- She has a strong experiential base as a NICU and transport nurse and has built her dissertation research on these experiences.

Weaknesses

- None noted.

2. Sponsors, Collaborators, and Consultants

Strengths

- There is an exceptional team of mentors with complementary expertise and strong commitment to the applicant. All are enthusiastic about the applicant and her research.

Weaknesses

- None noted.

3. Research Training Plan.

Strengths

- A strength is her research assistantship with Dr. Dail's NIH funded study.
- Her coursework is completed
- Her plan for dissertation defense is December 2023-May 2024.
- Attention to SDoH
- Unexplored area of research

Weaknesses

- The study timeline is for 18 months, completing the study data analysis in one year, but the timeline is not practical as the timeline provided is from 12/2022 to 12/2023.
- Completion of the study in essentially one year seems optimistic.
- There are few structured trainings planned, other than meetings with mentors.

- A lack of structured training in Mixed Methods is noted previously, and a Spring, 2023 short course is planned, but most of the training is self-directed.
- Sex as biological variable is not directly studied, although the genders will be equal.

4. Training Potential.

Strengths

- Her coursework is completed.
- She is on campus regularly even though the program does not require it and meets weekly with Dr. Dail.

Weaknesses

- The training proposed is largely individualized experiences with her mentors, which is very valuable, but gaps in her skills may not be as evident, although it is likely that Dr. Dail will be able to discern these.
- Much of the needed training has already occurred.

5. Institutional Environment & Commitment to Training.

Strengths

- A strong institutional commitment is evidenced by letters of support
- Excellent research environment at the university.
- Strong support from the NICU where infants will be admitted.

Weaknesses

- None noted.

Protections for Human Subjects: Acceptable Risks and Adequate Protections

- informed consent and appropriate exclusions

Data and Safety Monitoring Plan (Applicable for Clinical Trials Only): Not Applicable (No Clinical Trials)

Inclusion Plans

- Sex/Gender: Distribution justified scientifically
- Race/Ethnicity: Distribution justified scientifically
- For NIH-Defined Phase III trials, Plans for valid design and analysis: Not applicable
- Inclusion/Exclusion Based on Age: Distribution justified scientifically
- only Black infants, equal male and female

Vertebrate Animals: Not Applicable (No Vertebrate Animals)

Biohazards: Not Applicable (No Biohazards)

Resubmission

- Attentive to previous review

Training in the Responsible Conduct of Research: Acceptable

Comments on Format (Required):

- CITI training and coursework

Comments on Subject Matter (Required):

- inclusive

Comments on Faculty Participation (Required):

- Dr. Dail and Felder

Comments on Duration (Required):

- throughout program

Comments on Frequency (Required):

- not possible to assess accurately but appears every year there are activities

Resource Sharing Plans: Not Applicable (No Relevant Resources)

Authentication of Key Biological and/or Chemical Resources: Not Applicable (No Relevant Resources)

Budget and Period of Support: Recommend as Requested

CRITIQUE 3

Fellowship Applicant:	1
Sponsors, Collaborators, and Consultants:	3
Research Training Plan:	3
Training Potential:	3
Institutional Environment & Commitment to Training:	1

Overall Impact/Merit: This is a resubmission from a strong applicant whose research interests are aligned with her mentor's and are also based on a strong clinical background of vulnerability of neonates. The applicant has been responsive to prior critiques and strengthened the application by having instruments well described and improving the training in quantitative data analysis. The application has also been strengthened by focusing only on Black transported infants and their parents. The number of surveys to be completed by both parents in this very stressful time is not clear. There are a few methodical issues to be resolved, but the responsiveness of the applicant and the strong mentorship team and community support, are strong aspects of this application.

1. Fellowship Applicant

Strengths

- The applicant has an extensive clinical background to serve as a basis for her research interests. She has a strong academic record and sufficient prior research experience to be successful in this fellowship.
- The applicant has a reasonable chance of becoming an independent researcher. Her plans for a post doc after completion of her doctoral program are not set, however she is participating in a program that transitions doctoral students into tenure-track assistant professor positions.
- While the application builds on her clinical experience and aligns with her mentor's research program, the project is original and reflects her own scientific interest.
- The applicant has completed all of her coursework and has presented several posters on previous work as a research assistant and manuscripts are in preparation.

Weaknesses

- No major weaknesses noted.

2. Sponsors, Collaborators, and Consultants

Strengths

- There appears to be outstanding alignment between the sponsor's research qualifications and the needs of the applicant.
- The close association between the mentor and sponsor appears to reflect Dr. Dail's understanding of the applicant's needs. Dr. Dail has light doctoral mentorship responsibilities and should be able to work closely and frequently with the applicant. She has previously mentored a doctoral student in case study approach and mixed methods.
- There is a modest amount of research funding available to support the proposed dissertation activities. Her sponsor's R01 ends in the fall of 2023, however the team structure of her mentorship team and their roles are well defined.
- The applicant has the potential to receive excellent training and support from her team and should be able to move towards establishing a publication record. She is already engaged in national meetings and presenting posters.

Weaknesses

- The mentor's sources of research funding will be expiring in 2023. An update is needed to assure that the team has sufficient research resources to support the applicant.

3. Research Training Plan

Strengths

- The research proposed is well integrated with the research training plan and builds on the applicant's clinical strengths. The applicant has identified a significant, understudied area but her mentor's research program aligns well and is rigorous.
- The applicant's research plan is distinctly different from her mentors and is appropriate for a doctoral dissertation.
- The time frame appears sufficient to accomplish the study aims.
- The design has been revised to focus only on black neonates and their families.
- The study will prepare the applicant to transition to the next stage of her research career. She will bring a unique aspect to a team at the University of South Carolina (USC) that is focus on

neonatal care and health disparities. After this descriptive study of black families and their neonates she can easily transition to comparison studies with other ethnic/racial groups.

Weaknesses

- The applicant has strong ties with the partnering tertiary care center. It is not clear to what extent the depth of the electronic health record (EHR) from the transporting small hospitals will be. Nor is it really clear to what extent the quality of the transporting hospital will influence the experience being studied. It is unclear if the entire EHR from the transporting hospital will be available?
- The applicant speaks of interviewing both parents in the study. It is unclear to what extent the applicant expects the neonates to have two parents available for interview or what plans are in place if the data are based on only the mother's perceptions.

4. Training Potential

Strengths

- The proposed training plan and project addresses an understudied area and if accomplished will give the applicant favorable skills to advance her research career.
- Given the applicant's rich clinical experience and the training proposed, she will have the skills she needs to complete the work.
- The work will provide a valuable foundation upon which she can build more skill in other methods that will help her capture more effectively the inequities that may influence the outcomes of preterm transported infants and their families.

Weaknesses

- The content and quality of the nurse webinars completed in summer 2022 is difficult to assess.
- Given that all coursework is finished, and poster presentations have been done, would have better to see more focused training on dissemination through publications.

5. Institutional Environment & Commitment to Training

Strengths

- The partnership with Prisma Health Richland is essential for the execution of the project. A letter of support (LOS) is provided.
- The applicant has combined resources available at USC and other online training opportunities.

Weaknesses

- None noted.

Protections for Human Subjects: Acceptable Risks and Adequate Protections

- The applicant has been responsive to prior critique but participation of the study by both parents at multiple times could be burdensome.
- It's not clear with $25 per survey what will be the minimum and maximum a parent could receive. It is unclear if father and mother will receive separate reimbursement if it is not an intact family.

Data and Safety Monitoring Plan (Applicable for Clinical Trials Only): Not Applicable (No Clinical Trials)

Inclusion Plans

- Sex/Gender: Distribution justified scientifically
- Race/Ethnicity: Distribution justified scientifically
- For NIH-Defined Phase III trials, Plans for valid design and analysis: Not applicable
- Inclusion/Exclusion Based on Age: Distribution justified scientifically

Vertebrate Animals: Not Applicable (No Vertebrate Animals)

Biohazards: Not Applicable (No Biohazards)

Resubmission

- The applicant has made major changes in the design of the research project. Other strengths noted in the past review are still present.

Training in the Responsible Conduct of Research: Acceptable

Comments on Format (Required):

- A combination of content from doctoral courses she has taken and the CITI training.

Comments on Subject Matter (Required):

- Subject areas are covered sufficiently.

Comments on Faculty Participation (Required):

- Faculty involvement is high in the subjects covered in the curriculum

Comments on Duration (Required):

- Seem adequate

Comments on Frequency (Required):

- CITI training is repetitive. Little information on refreshers from content covered in classes.

Resource Sharing Plans: Not Applicable (No Relevant Resources)

Authentication of Key Biological and/or Chemical Resources: Not Applicable (No Relevant Resources)

Budget and Period of Support: Recommend as Requested

THE FOLLOWING SECTIONS WERE PREPARED BY THE SCIENTIFIC REVIEW OFFICER TO SUMMARIZE THE OUTCOME OF DISCUSSIONS OF THE REVIEW COMMITTEE, OR REVIEWERS' WRITTEN CRITIQUES, ON THE FOLLOWING ISSUES:

PROTECTION OF HUMAN SUBJECTS: ACCEPTABLE

INCLUSION OF WOMEN PLAN: ACCEPTABLE

INCLUSION OF MINORITIES PLAN: ACCEPTABLE

INCLUSION ACROSS THE LIFESPAN: ACCEPTABLE

TRAINING IN THE RESPONSIBLE CONDUCT OF RESEACH: ACCEPTABLE

COMMITTEE BUDGET RECOMMENDATIONS: The budget was recommended as requested.

Footnotes for 1 F31 NR020731-01A1; PI Name: Warren, Karen F

NIH has modified its policy regarding the receipt of resubmissions (amended applications).See Guide Notice NOT-OD-18-197 at https://grants.nih.gov/grants/guide/notice-files/NOT-OD-18-197.html. The impact/priority score is calculated after discussion of an application by averaging the overall scores (1-9) given by all voting reviewers on the committee and multiplying by 10. The criterion scores are submitted prior to the meeting by the individual reviewers assigned to an application, and are not discussed specifically at the review meeting or calculated into the overall impact score. Some applications also receive a percentile ranking. For details on the review process, see http://grants.nih.gov/grants/peer_review_process.htm#scoring.

MEETING ROSTER

National Institute of Nursing Research Initial Review Group
NATIONAL INSTITUTE OF NURSING RESEARCH
NRRC (86)
02/23/2023 - 02/24/2023

Notice of NIH Policy to All Applicants: Meeting rosters are provided for information purposes only. Applicant investigators and institutional officials must not communicate directly with study section members about an application before or after the review. Failure to observe this policy will create a serious breach of integrity in the peer review process, and may lead to actions outlined in NOT-OD-22-044 at https://grants.nih.gov/grants/guide/notice-files/NOT-OD-22-044.html, including removal of the application from immediate review.

CHAIRPERSON(S)

NELSON, LARON EARNEST, MS, PHD
ASSOCIATE PROFESSOR AND ASSOCIATE DEAN
GLOBAL AFFAIRS AND PLANETARY HEALTH
SCHOOL OF NURSING
YALE UNIVERSITY
NEW HAVEN, CT 06520

MEMBERS

BADR, HODA J, MA, PHD *
ASSOCIATE PROFESSOR
DEPARTMENT OF MEDICINE
BAYLOR COLLEGE OF MEDICINE
HOUSTON, TX 77030

BURCH, JAMES, PHD, MS *
PROFESSOR
DIVISION OF EPIDEMIOLOGY
FAMILY MEDICINE AND POPULATION HEALTH DEPARTMENT
VIRGINIA COMMONWEALTH UNIVERSITY
RICHMOND, VA 23219

FOWKE, JAY H, PHD, MPH *
PROFESSOR
DEPARTMENT OF PREVENTIVE MEDICINE
COLLEGE OF MEDICINE - MEMPHIS
THE UNIVERSITY OF TENNESSEE HEALTH SCIENCE CENTER
MEMPHIS, TN 38163

GROER, MAUREEN EDITH, PHD, RN *
PROFESSOR
COLLEGE OF NURSING
UNIVERSITY OF SOUTH FLORIDA
TAMPA, FL 33612

HAPP, MARY E, PHD, FAAN
PROFESSOR AND ASSOCIATE DEAN FOR RESEARCH AND INNOVATION
COLLEGE OF NURSING
THE OHIO STATE UNIVERSITY
COLUMBUS, OH 43210

KNEIPP, SHAWN M., PHD
PROFESSOR,
SCHOOL OF NURSING
UNIVERSITY OF NORTH CAROLINA AT CHAPEL HILL
CHAPEL HILL, NC 27516

KOHLER, PAMELA, PHD *
ASSOCIATE PROFESSOR
DEPARTMENT OF CHILD, FAMILY,
AND POPULATION HEALTH NURSING
UNIVERSITY OF WASHINGTON
SEATTLE, WA 98105

LYONS, KATHLEEN DOYLE, BS, MS, SCD *
SENIOR SCIENTIST
PROFESSOR
OCCUPATIONAL THERAPY
MGH INSTITUTE OF HEALTH PROFESSION
BOSTON, MA 02129

MCCAULEY, LINDA A., PHD, MSN, RN, FAAN
DEAN AND PROFESSOR
NELL HODGSON WOODRUFF SCHOOL OF NURSING
EMORY UNIVERSITY
ATLANTA, GA 30322

PARKER, LESLIE A, PHD *
PROFESSOR
COLLEGE OF NURSING
UNIVERSITY OF FLORIDA
GAINESVILLE, FL 32610

PHILLIPS II, GREGORY L, PHD, MS *
ASSISTANT PROFESSOR
MEDICAL SOCIAL SCIENCES AND PREVENTIVE MEDICINE
FEINBERG SCHOOL OF MEDICINE
NORTHWESTERN UNIVERSITY
625 N MICHIGAN AVE, SUITE 2700
CHICAGO, IL 60611

RAWL, SUSAN M, PHD, FAAN
PROFESSOR
SCHOOL OF NURSING
INDIANA UNIVERSITY
INDIANAPOLIS, IN 46202

RODRIGUEZ, ERIN M., PHD
ASSOCIATE PROFESSOR AND PROGRAM CHAIR
DEPARTMENT OF EDUCATIONAL PSYCHOLOGY
UNIVERSITY OF TEXAS AT AUSTIN
AUSTIN, TX 78712

SAWYER, AMY M, MSN, PHD, RN *
ASSOCIATE PROFESSOR
SCHOOL OF NURSING
UNIVERSITY OF PENNSYLVANIA
PHILADELPHIA, PA 19104

SPRUILL, TANYA M, MS, PHD *
ASSOCIATE RESEARCH SCIENTIST
DEPARTMENTS OF POPULATION HEALTH AND MEDICINE
NEW YORK UNIVERSITY SCHOOL OF MEDICINE
NEW YORK, NY 10032

TALEGAWKAR, SAMEERA A, PHD *
ASSOCIATE PROFESSOR
DEPARTMENT OF EXERCISE AND NUTRITION SCIENCES
MILKEN INSTITUTE SCHOOL OF PUBLIC HEALTH
THE GEORGE WASHINGTON UNIVERSITY
WASHINGTON, DC 20052

TRENT, MARIA E., MPH, MD, BA
PROFESSOR AND CHIEF
DIVISION OF ADOLESCENT MEDICINE
DEPARTMENT OF PEDIATRICS
JOHNS HOPKINS SCHOOL OF MEDICINE
BALTIMORE, MD 21287

WOO BAIDAL, JENNIFER AIMEE, MD, MPH
ASSISTANT PROFESSOR
DEPARTMENT OF PEDIATRICS
DIVISION OF PEDIATRIC GASTROENTEROLOGY,
HEPATOLOGY AND NUTRITION
COLUMBIA UNIVERSITY IRVING MEDICAL CENTER
NEW YORK, NY 10032

SCIENTIFIC REVIEW OFFICER

LI, WEIQUN, MD
SCIENTIFIC REVIEW OFFICER
OFFICE OF REVIEW
NATIONAL INSTITUTE OF NURSING RESEARCH
NATIONAL INSTITUTES OF HEALTH
6701 DEMOCRACY BLVD., SUITE 710
BETHESDA, MD 20892

OTHER REVIEW STAFF

BHATTACHARYYA, NISAN, PHD
SCIENTIFIC REVIEW OFFICER
OFFICE OF SCIENTIFIC REVIEW
NATIONAL INSTITUE OF NURSING RESEARCH
NATIONAL INSTITUTES OF HEALTH
BETHESDA, MD 20892

RIZAK, KATHLEEN O
EXTRAMURAL SUPPORT ASSISTANT
1DEM RM 715 6701 DEMOCRACY BLVD
EXTRAMURAL SUPPORT ASSISTANT
BETHESDA, MD 20817

YAN, MING, MD, PHD
SCIENTIFIC REVIEW OFFICER
OFFICE OF SCIENTIFIC REVIEW
NATIONAL INSTITUTE OF NURSING RESEARCH
NATIONAL INSTITUTES OF HEALTH
BETHESDA, MD 20892

* Temporary Member. For grant applications, temporary members may participate in the entire meeting or may review only selected applications as needed.

Consultants are required to absent themselves from the room during the review of any application if their presence would constitute or appear to constitute a conflict of interest.
